# Structural Paradigms in the Recognition of the Nucleosome Core Particle by Histone Lysine Methyltransferases

**DOI:** 10.3389/fcell.2020.00600

**Published:** 2020-07-31

**Authors:** Ashley Janna, Hossein Davarinejad, Monika Joshi, Jean-Francois Couture

**Affiliations:** Ottawa Institute of Systems Biology, Shanghai Institute of Materia Medica-University of Ottawa Research Center in Systems and Personalized Pharmacology, Department of Biochemistry, Microbiology and Immunology, University of Ottawa, Ottawa, ON, Canada

**Keywords:** histone, epigenetics, methylation, ubiquitinylation, chromatin

## Abstract

Post-translational modifications (PTMs) of histone proteins play essential functions in shaping chromatin environment. Alone or in combination, these PTMs create templates recognized by dedicated proteins or change the chemistry of chromatin, enabling a myriad of nuclear processes to occur. Referred to as cross-talk, the positive or negative impact of a PTM on another PTM has rapidly emerged as a mechanism controlling nuclear transactions. One of those includes the stimulatory functions of histone H2B ubiquitylation on the methylation of histone H3 on K79 and K4 by Dot1L and COMPASS, respectively. While these findings were established early on, the structural determinants underlying the positive impact of H2B ubiquitylation on H3K79 and H3K4 methylation were resolved only recently. We will also review the molecular features controlling these cross-talks and the impact of H3K27 tri-methylation on EZH2 activity when embedded in the PRC2 complex.

## Introduction—the Nucleosome

The genetic material of a typical eukaryotic cell approximately measures 2 meters and must be restricted to the confines of the nucleus. The cell employs four α-helical basic proteins to create a scaffold around which DNA can be compacted: histones H2A, H2B, H3, and H4. First, two histone H3–H4 heterodimers dimerize to form a heterotetramer, upon which two H2A–H2B heterodimers will bind. The H2A protomers contact H3 and H4 at the extremities of the heterotetramer; meanwhile, the H2B protomers form an extensive dimerization interface (Arents et al., 1991) to create a symmetrical disk-shaped histone octamer. A DNA fragment of approximately 150 bp will then wrap twice around the histone octamer of basic histone proteins to form a repetitive structure known as the nucleosome (Noll, 1977; Luger et al., 1997) [referred therein as nucleosome core particle (NCP)]. However, in recent years, incorporation of histone variants in nucleosomes brought diversity to that model (Koyama and Kurumizaka, 2018; Talbert et al., 2019).

## Lysine Methylation

Protein lysine methylation involves the transfer of up to three methyl groups to the ϵ-amine of a lysine residue. To this day, lysine methylation has been observed in both nuclear and cytoplasmic proteins and is now considered a prevalent modification in eukaryotes, prokaryotes, and archaea (Iwabata et al., 2005; Jung et al., 2008; Botting et al., 2010; Pang et al., 2010). Methylation of a lysine residue was first reported by Ambler and Rees (1959) in the flagellin protein of *Salmonella typhimurium*. These findings, further led by additional studies on histone H1, H3, and H4 lysine methylation (Couture and Trievel, 2006; Lee et al., 2010), unveiled that this post-translational modification (PTM) fine-tunes the activity of transcription factors (Yang et al., 2009), participates in the assembly of multi-subunit complexes (Zhang et al., 2005; Donlin et al., 2012), and contributes to the structural organization of chromosomes (Lanouette et al., 2014).

## Histone Lysine Methylation; When Plants Provide the First Hint

Initially reported by Allfrey et al. (1964), the field of histone lysine methylation grew exponentially in the early 2000 after the identification that the Large Subunit MethylTransferase (LSMT) can methylate lysine 14 of Ribulose-1,5-bisphosphate carboxylase/oxygenase (Ying et al., 1999). Following this seminal discovery, the group of Thomas Jenuwein reported the methylation of Lys-9 on histone H3 by the SUV3/9 family of methyltransferases (MTs; Rea et al., 2000). During the same period, using basic alignment tools, several groups identified evolutionary conserved motifs (GXG, YXG, NHXCXPN) found in a wide range of evolutionary conserved proteins (Jenuwein, 2001). Given the enrichment of these motifs in proteins including Suppressor of variegation, Enhancer of zeste, and Trithorax (SET) (Jenuwein, 2001), these enzymes were coined as SET domain lysine MTs. However, over the years, few notable cases of histone lysine MTs, such as Dot1 and PR domain MTs (PRDM), were reported to lack a SET domain. Therefore, the nomenclature for these enzymes was changed to lysine (K) MT (Allis et al., 2007). Since their discoveries, these enzymes have been shown to site-specifically methylate histone and non-histone substrates and are now recognized as critical regulators of chromatin structure and other cellular functions (Lanouette et al., 2014). They are extremely specific and, in most cases, have the ability to recognize a single lysine side chain on a single protein (Lanouette et al., 2014).

## Different Mechanisms of Histone Recognition and Methylation by Set Domain HKMTs

Despite being evolutionary conserved, SET domain HKMTs can be separated into at least two different categories. This classification arises from many studies showing that HKMTs display divergence in their catalytic properties when homogeneously purified. For example, the histone H3 K36 MT SETD2 methylates, with the same catalytic efficiency (Eram et al., 2015), a peptide, the full-length histone H3 or the NCP. Conversely, other HKMTs such as ATXR5/6, EZH2, and SET8 preferentially methylate the NCP (Nishioka et al., 2002; Kirmizis et al., 2004; Margueron et al., 2008; Qiao et al., 2011). These observations suggest that this subgroup of SET domain HKMTs harbor unique structural determinants able to bind DNA. Moreover, the ubiquitination of the nucleosome or chromatin template creates better substrates for Dot1 and SET1 enzymes, respectively. Recently, several cryo-EM structures unraveled the intricacies underlying the recognition of the nucleosome by the EZH2 complex and the ubiquitinated form of the nucleosome by Dot1 and members of the SET1 family of MTs. Below, we will review the critical observations reported in these papers.

## Structural Insights Into the Recognition of H2Bub Nucleosome by Dot1L

Initially identified in a genetic screen to discover genes conferring defects in telomeric silencing (Singer et al., 1998; Nguyen and Zhang, 2011), disruptor of telomeric silencing-1 (Dot1) remained, for several years, the only non-SET domain histone lysine MTs. Biochemical characterization of Dot1 revealed that the enzyme mono-, di-, or tri-methylate H3K79, a modification initially linked to transcriptional regulation and DNA damage response (Nguyen and Zhang, 2011). Evolutionary conserved (Feng et al., 2002; Vlaming and van Leeuwen, 2016), human Dot1L is composed of 1537 highly conserved residues. The catalytic site is located on the N-terminus of the protein while its C-terminal extension interacts with proteins that direct Dot1L to specific genomic loci (Kuntimaddi et al., 2015; Worden et al., 2019). Initial biochemical characterization of Dot1L revealed that the MT activity of Dot1L depends on two critical factors. First, Dot1L prefers to methylate H3K79 in the context of the nucleosome (Feng et al., 2002; McGinty et al., 2008). Second, mono-ubiquitination of histone H2B on lysine 120 (Briggs et al., 2002; Ng et al., 2002; McGinty et al., 2008) (H2BK120ub) greatly enhances H3K79 methylation. Initial model showing that H2BK120 and H3K79 are closely juxtaposed on the same solvent-exposed surface of the mono-nucleosome (McGinty et al., 2008; Wood et al., 2018; Zhang and Kutateladze, 2019) lend further credence to that model. However, despite important structural insights provided by the crystal structure of Dot1L catalytic domain (Min et al., 2003), the molecular underpinnings underlying the positive impact of H2B ubiquitination on K79 methylation by Dot1L remained unexplained. Recently, several structures provided insights into the various steps linked to Dot1L binding to (Anderson et al., 2019; Worden et al., 2019; Yao et al., 2019), methylation of (Worden et al., 2019), and disengagement from (Valencia-Sanchez et al., 2019) the nucleosomes. Three steps referred to as poised, active, and post-catalysis states.

## Dot1L Recognizes H2A–H2B Acidic Patch in the Nucleosome Via an Arginine Anchor

**FIGURE 1:**
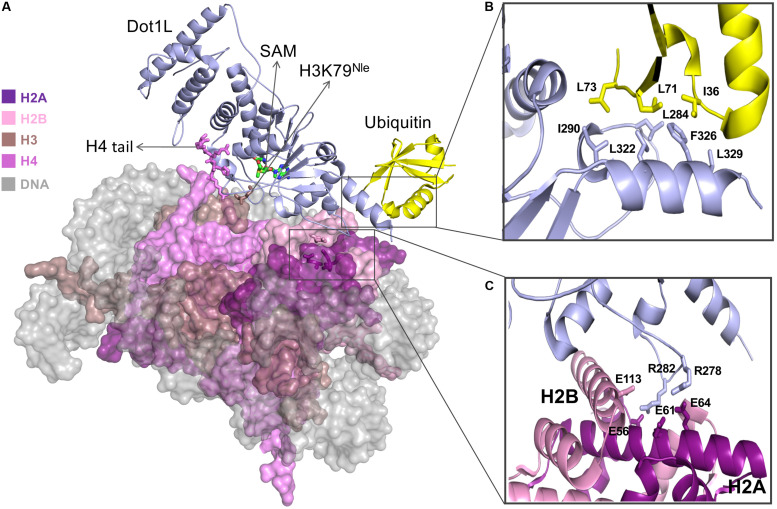
**(A)** Cryo-EM structure of Dot1L bound to H2B ubiquitinated nucleosome in active state. SAM cofactor, H4 tail, H3K79^Nle^ and H2A-H2B acidic patch residues are depicted in stick model and nucleosome core particle (NCP) is depicted in surface representation **(B)** Detailed view of interaction between Dot1L and ubiquitin. Important residues at the Dot1L-ubiqutin interface are shown as sticks **(C)** Close-up of residues interactions between Dot1L and H2A-H2B acidic patch. Figures are generated using the cryo-EM structure of the Dot1L bound to H2B-Ubiquitin Nucleosome complex in active state (PDB accession number 6NJ9; Worden et al., 2019).

Initial biochemical studies revealed that Dot1L preferentially methylates K79 when histone H3 is embedded in the NCP. The cryo-EM structures of Dot1L show that the C-terminal region of Dot1L contacts ubiquitin and the acidic patch of H2A–H2B (Figure 1). In the C-terminal region of Dot1L, a long nucleosome-interacting loop, which connects two parallel β-strands, makes contacts with the acidic patch on the nucleosome (Valencia-Sanchez et al., 2019; Worden et al., 2019). More specifically, this loop contains two evolutionary conserved arginine residues (Arg278 and Arg282) that recognize the H2A–H2B acidic patch on the nucleosome (Anderson et al., 2019; Jang et al., 2019; Valencia-Sanchez et al., 2019; Worden et al., 2019; Yao et al., 2019) (Figure 1). Interestingly, these structures show that, akin to SIR3 (Armache et al., 2011), latency-associated nuclear antigen (Barbera et al., 2006), RCC1 (Makde et al., 2010), PRC1 Ubiquitylation Module (McGinty et al., 2014), Dot1L uses arginine anchors to engage the H2A–H2B acidic patch located on the surface of the nucleosomal disk.

The active site of Dot1L, consisting of an S-adenosyl-L-methionine (SAM) binding pocket and a lysine-binding channel, is positioned above H3K79 in the cryo-EM structure of the DOT1L-H2BK120Ub nucleosome complex (poised state—see below). Three loops of Dot1L form the lysine-binding channel that connects the side chain of H3K79 to the methyl donor SAM. Within these loops, several aromatic and hydrophobic residues surround the entrance of the channel and make direct contact with H3 residues adjacent to K79 (Yao et al., 2019). Within the complex, the histone H4 tail sits on α2 helix of histone H3 and extends to the N-terminal region of Dot1L and its active site to mediate extensive electrostatic and hydrophobic interactions with the MT. The importance of this network of interaction is underscored by mutational studies showing that substitution of histone H4 residues negatively impact the methylation of K79 by Dot1L (Yao et al., 2019).

## Dot1L Recognizes H2B Ubiquitin via Its Hydrophobic C-Terminal Helix

The cryo-EM structure of Dot1L–H2BK120Ub nucleosome complex reveals that Dot1L extensively interacts with core histones on the disk-face of nucleosome with its C-terminal region sandwiched between ubiquitin and the histone H2A–H2B dimer (Figure 1). The direct association of Dot1L with the H2BK120-conjugated ubiquitin extends the recognition interface between Dot1L and the surface of the NCP. Docking of the Dot1L–H2BK120Ub nucleosome complex cryo-EM structure with the cryo-EM structure of Dot1L in complex with an unmodified nucleosome complex shows a good fit of the Dot1L–H2BK120Ub nucleosome complex structure with the Dot1L-unmodified nucleosome complex, indicating that mono-ubiquitination of H2BK120 does not change the overall location of Dot1L on the surface of the nucleosome (Yao et al., 2019). The structures show the proximity of H2B-ubiquitin and the C-terminal helix of the Dot1L catalytic domain. A hydrophobic patch on ubiquitin lies near several hydrophobic residues located on an alpha helical region of Dot1L. More specifically, an area surrounding Ile36 on ubiquitin stacks on a hydrophobic patch surrounding Phe326 on Dot1L (Figure 1). The importance of these interactions was confirmed by mutational studies followed by histone MT assays which showed that substitution of these hydrophobic residues impairs H3K79 methylation activity of Dot1L toward ubiquitinated nucleosome but has a minor impact on the ability of Dot1L to methylate the unmodified nucleosome (Anderson et al., 2019; Jang et al., 2019; Valencia-Sanchez et al., 2019; Worden et al., 2019; Yao et al., 2019).

## Cryo-EM Studies of Dot1L Unravels Three States

Comparative analysis of Dot1L structures bound to the ubiquitinated form of the nucleosome revealed three structurally distinct forms of the complex. In the first form, also referred to as the poised state, Dot1L is positioned above histone H3K79. In this conformation, Dot1L makes contacts with ubiquitin and adjacent regions of H3K79 (Yao et al., 2019) as well as uses its arginine residues to bind to the NCP acidic patch. The observation that the catalytic site of Dot1L is separated from H3K79 indicates that Dot1L and/or the nucleosome must undergo conformational rearrangement from a poised to an active state to enable methylation (Anderson et al., 2019; Jang et al., 2019; Valencia-Sanchez et al., 2019; Worden et al., 2019; Yao et al., 2019). To trap the active state, the Cryo-EM structure of Dot1L was solved in complex with a modified ubNCP wherein K79 on histone H3 is replaced by Norleucine (Nle) (Figure 1A) (Worden et al., 2019; Zhang and Kutateladze, 2019); a non-native amino acid that increases the affinity of lysine MTs for their substrates in a cofactor-dependent manner (Brown et al., 2014; Jayaram et al., 2016).

Trapping the active state of the complex enabled the following observations. First, ubiquitin on H2BK120 notably restricts the orientation of Dot1L in the complex, forcing the active site of Dot1L to face the nucleosome. The contact between Dot1L and the H2A-H2B acidic patch further limits Dot1L’s motion, positioning Dot1L in a catalytically competent orientation. In both active and poised state complexes, Dot1L C-terminus contacts ubiquitin and the nucleosome acidic patch, anchoring Dot1L to one edge of the nucleosome and therefore providing a pivot point about which Dot1L can rotate. The active state is further stabilized by an interaction between the histone H4 tail and a groove located in the N-terminal region of Dot1L, a region situated ∼5 Å away from the pivot contact point, but brings another N-terminal part of Dot1L closer to the nucleosome surface. Compared to the poised state, the active state of Dot1L is rotated clockwise by ∼20° around the ubiquitin and pivots down toward the nucleosome face by 25 Å (Worden et al., 2019; Zhang and Kutateladze, 2019). Interestingly, the side chain of K79 of histone H3 in the poised state complex is inaccessible for catalysis, lying parallel to the lateral surface of the nucleosomal histone core. However, in the active state, a conformational change of K79^Nle^ and its neighboring residues reorients both its backbone and side chain by ∼90°. This movement exposes K79 ε-amine to the solvent and enables its insertion into Dot1L active site (Worden et al., 2019; Zhang and Kutateladze, 2019).

The post-catalysis state was determined in the presence of S-adenosyl homocysteine and, as evidenced by mass spectrometry, H3K79 mono- and di-methylated NCP (Valencia-Sanchez et al., 2019). In this conformation, the distance between Dot1L active site and H3K79 is approximately 22 Å and unlike the poised state structure (Anderson et al., 2019; Jang et al., 2019; Yao et al., 2019), the post-catalysis state of Dot1L maintains interactions with the histone H4 tail (Valencia-Sanchez et al., 2019). Overall, the post-catalysis structure shows that Dot1L establishes multivalent interactions on the surface of the nucleosome including histone H4 tail and H2A–H2B acidic patch in addition to ubiquitin.

However, distortion of the cryo-EM density map of Dot1L’s C-terminal helix suggests that motion at this site and near the acidic patch is reduced by ubiquitin. This facilitates Dot1L to carry mono-methylation, and even di- and tri-methylation of H3K79 irrespective of H2B ubiquitination. Collectively, cryo-EM structures of Dot1L in complex with ubiquitinated nucleosome complemented with biochemical experiments provided critical insights into the molecular mechanism of Dot1L-mediated methylation of lysine 79 in histone H3 and explained its crosstalk with histone H2B ubiquitination.

## Recognition of H2B Ubiquitinated NCP by Compass

**FIGURE 2:**
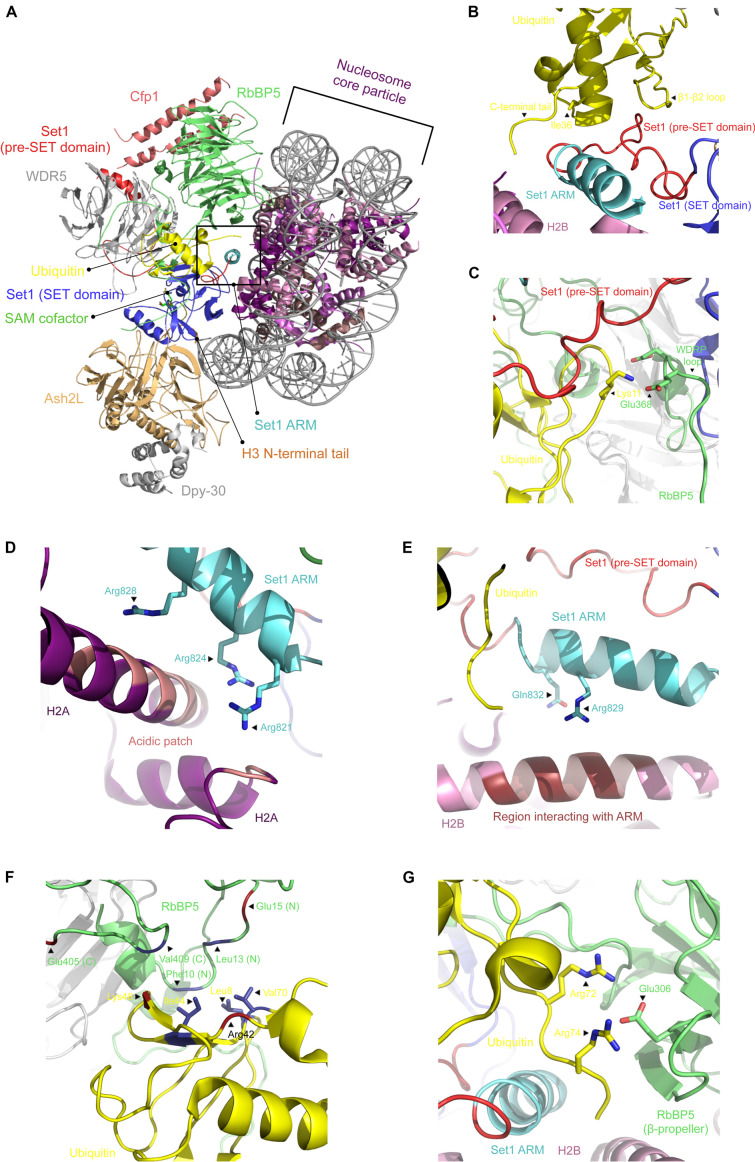
Cryo-EM structure of COMPASS bound to the ubiquitinated nucleosome core particle. **(A)** Cartoon representation of COMPASS cryo-EM structure bound to the ubiquitinated nucleosome in which each subunit is indicated. **(B)** Zoomed view on the interactions between ubiquitin and the pre-SET domain of SET1. Shown are the polar contacts between RbBP5 WDRP loop and ubiquitin **(C)** and the cluster of positively charged residues of the SET1 ARM motif interacting with the H2A acidic patch **(D)** and histone H2B **(E)**. Cartoon representation of RbBP5 N- and C-termini that make contacts with ubiquitin’s hydrophobic patch (depicted as sticks) **(F)**, as well as the contacts made between its β-propeller domain and ubiquitin C-terminal end **(G)**. All figures were prepared using the cryo-EM structure of the COMPASS catalytic module in complex with the ubiquitinated nucleosome (PDB accession number 6UH5).

Initially identified in yeast, the complex associated with SET1 (COMPASS) is formed of several regulatory subunits including WDR5, RbBP5, Ash2L, DPY-30, CFP1, BIG1, as well as the catalytic unit SET1 (Miller et al., 2001). Each subunit plays important roles in the biology of SET1 and contributes, to various extents, to the H3K4 MT activity of the complex. Owing to its link to various aggressive forms of cancers (Ford and Dingwall, 2015; Rao and Dou, 2015), several groups elucidated the crystal structure of several subunits including WDR5 (Patel et al., 2008; Dharmarajan et al., 2012; Zhang et al., 2012), RbBP5 (Mittal et al., 2018; Han et al., 2019), Ash2L (Chen et al., 2011, 2012; Sarvan et al., 2011; Zhang et al., 2015), the catalytic domain of SET1 (or its homologs) (Malumbres et al., 1997; Li et al., 2016), as well as Cfp1 (Xu et al., 2011; He et al., 2019; Yang et al., 2020). While these structures provided critical insights into the molecular underpinnings controlling the formation of COMPASS, they did not capture the entire spectrum of interactions contributing to the assembly of COMPASS. The first glimpse at COMPASS assembly was unraveled by the cryo-EM structure of budding yeast COMPASS (Qu et al., 2018) and the crystal structure of the SET1 catalytic module (Hsu et al., 2018). The cryo-EM structure shows that COMPASS assembles in a Y-shaped conformation in which WDR5 and RbBP5 (Cps30 and Cps50) β-propeller domains form the upper tips of COMPASS. Cfp1 (Cps40) connects these propellers, while Ash2L (Cps60) and Dpy-30 (Cps25) form the base of the complex. The catalytic domain of SET1 is found at the junction of the Y-shaped complex and makes contacts with every subunit, except for Dpy-30 (Qu et al., 2018) (Figure 2A). Interestingly, the cryo-EM structure nicely explains the modest stimulatory functions of Dpy-30 on the MT activity of SET1 on peptides when the complex is assembled with purified components (Haddad et al., 2018). Clustering of the particles revealed two conformationally distinct complexes, suggesting that COMPASS is a structurally dynamic complex that can exist in at least two conformers likely helping COMPASS to adapt to the structurally dynamic environment of chromatin (Maeshima et al., 2019).

H3K4 methylation by COMPASS is stimulated when the nucleosome is ubiquitinated on H2B (Sun and Allis, 2002; Kim et al., 2013; Holt et al., 2015). Recently, several papers documented the structural details controlling the recognition of the ubiquitinated form of the nucleosome. These structures show that COMPASS recognizes two parts of the nucleosome. On the one hand, COMPASS binds the surface of the NCP disk and the first eight residues of histone H3. Except for WDR5 and DPY-30, all the other subunits directly contact the histone proteins, ubiquitin, and/or the nucleosomal DNA (Hsu et al., 2019). COMPASS engages both the ubiquitinated and non-ubiquitinated nucleosomes in similar fashions. However, in the presence of histone H2B ubiquitination, RbBP5 and SET1 make additional contacts with the ubiquitin moiety (Figures 2B–G). The SET1 catalytic domain packs against the H2A α2 helix using two points of contact. First, a region preceding the SET1 catalytic domain contacts three residues on H2A. The same protein also surrounds the C-terminus of the same helix on H2A using a cluster of five evolutionarily conserved hydrophobic residues. The presence of this cluster in other members of the KMT2 family of enzymes points to a model wherein the catalytic domain of these enzymes may bind similarly to the surface of the nucleosome. Correlatively, mutations of these residues result in a loss of H3K4 di- and tri-methylation (Nakanishi et al., 2008). In the presence of ubiquitinated H2B, a region immediately preceding the catalytic domain of SET1, which includes an Arginine Rich Motif (ARM), and a fraction of its SET domain form a coil binding to a pocket formed by Ile36, the β1–β2 loop, and the tail of ubiquitin (Figure 2B). The ARM motif, which is sandwiched between COMPASS subunits and uNCP, is located near the acidic patch created by the H2A–H2B interface (Nakanishi et al., 2008; Kim et al., 2013) (Figures 2D,E). These observations are supported by biochemical and *in vivo* data showing that mutation of the residues forming the ARM motif negatively impacts H3K4 methylation (Kim et al., 2013). Altogether, these observations indicate that this motif serves as an important link between H2B ubiquitination and H3K4 methylation.

Several hydrophobic residues located on both the N- and C-termini of RbBP5 interact with a hydrophobic patch on ubiquitin (Figure 2F). The β-propeller domain of RbBP5 also makes polar contacts with the C-terminus of ubiquitin (Figure 2G). In addition to binding to ubiquitin, RbBP5 directly interacts with a cleft formed by α3 and αC of histone H2B, α2 of H2A, as well as DNA. Mutation of the residues forming this RbBP5–NCP interface impair H3K4 methylation by COMPASS, underscoring the importance of these interactions (Hsu et al., 2019). Located in the same region of COMPASS and directly interacting with RbBP5 (Yang et al., 2020), weak but discernable structural information can be detected in a region of Cfp1 composed of positively charged residues. Based on the predicted location of these residues near the nucleosomal DNA, the cryo-EM structure suggests that Cfp1 directly binds DNA. Similarly, the Ash2L (Cps60) SPRY domain directly interacts with the phosphate backbone of the nucleosomal DNA (Hsu et al., 2019).

The cryo-EM structures of COMPASS in complex with the ubiquitinated and non-ubiquitinated nucleosomes have provided important information regarding how COMPASS engages its substrate and the structural underpinnings mediating its enzymatic activity. The findings suggest that the presence of ubiquitin may alter the dynamics of the catalytic subunit in alleviating an auto-inhibitory function of the SET1 ARM motif (Hsu et al., 2019). Furthermore, the interactions between COMPASS and uNCP appear to stabilize further the N-terminus of histone H3 in the catalytic domain. In the absence of ubiquitin, the structure presents only three H3 residues (T3, K4, and Q5) interacting with SET1, while in the presence of ubiquitin, A1 to R8 are distinguishable in the SET1 catalytic domain. This suggests that the interactions between COMPASS and ubiquitin induce conformational changes that increase the interface between the catalytic domain of SET1 and the residues flanking H3K4. Altogether, these results show that cross-talk between protein complex subunits and pre-existing modifications on the nucleosome represents a way to control H3K4 methylation (Jeon et al., 2018). Interestingly, such cross-talk has also been proposed as a mode of activation for other histone MTs, such as EZH2 (Margueron et al., 2009; Jiao and Liu, 2015; Brooun et al., 2016).

## EZH2 and H3K27Me3

The Enhancer of zeste E(z) gene was discovered as an important regulatory element in maintaining suppression of homeotic gene expression such as those determining pigmentation in *Drosophila melanogaster* (Kalisch and Rasmuson, 1974; Wu et al., 1989). A subsequent study revealed that the C-terminal region of E(z) gene product, now known as the SET domain, shares homology with regions of the Trithorax (Trx) (Jones and Gelbart, 1993) and Supressor of variegation [Su(var)] proteins. In humans, EZH2 is one of the two homologs of *the fruit fly’s* E(z) enzyme which trimethylates H3K27 and preferentially methylates dinucleosomes substrates over mononuclesomes, and the MT activity is further stimulated by the linker histone H1 (Martin et al., 2006). Local H3K27me3 is linked to suppression of targeted gene expression while this mark can spread to regulate processes such as cell differentiation and X-chromosome inactivation by negatively regulating gene expression. EZH2 SET domain is the catalytic component of Polycomb repressive complex 2 (PRC2) which also includes embryonic ectoderm development (EED), suppressor of zeste 12 (SUZ12), and Retinoblastoma Binding Protein 4 (RbBP4) as core components. In contrast to SET domain lysine MTs such as ATXR5/6 (Jacob et al., 2009), EZH2 alone is not catalytically active and minimally requires EED and the VEFS [Vrn2-Emf2-Fis2-Su(z)12] box of SUZ12 to methylate H3K27 (Cao and Zhang, 2004). Other components, namely, jumonji AT-rich interactive domain 2 (JARID2), Adipocyte Enhancer-Binding Protein 2 (AEBP2), and polycomb-like (PCL) proteins associate with and modulate PRC2 activity or its recruitment to chromatin. These include interaction with unmethylated CpG islands (Li et al., 2017), activation at *de novo* H3K27me3 nucleation sites (Oksuz et al., 2018), or determining exclusivity of PRC2 subcomplexes (Grijzenhout et al., 2016). Although earlier understanding of the relationship between PRC2 and PRC1, which monoubiquitinates K119 on H2A (Wang et al., 2004), suggested that cooperative repression by these complexes is mediated by the detection of H3K27me3 via Cbx in PRC1 (Senthilkumar and Mishra, 2009), recent evidence suggest that JARID2 also binds the H2A-K119ubiquitinated form of the NCP suggesting that cross-talk between PRC1 and PRC2 involves more than H3K27me3 and that it may not be unidirectional or in the chronological order previously described.

## Structural Analysis of PRC2 and EZH2 Activation by the Holoenzyme

The absence of EZH2 activity was elegantly explained by the crystal structure of EZH2 CXC-SET domains alone (Wu et al., 2013). The structure shows that the EZH2 substrate-binding groove is in a closed state as a result of hydrogen bonds between residues in the I-SET and post-SET regions of EZH2 likely barring the H3K27 to enter the channel. The CXC domain also appears to play an autoinhibitory role in EZH2 by pulling away from the post-SET domain, which contributes to the formation of the cofactor binding site rendering this pocket structurally incomplete. The crystal structure of the minimal PRC2 complex revealed an extensive network of inter-domain interactions involving all domains of EZH2, EED, and VEFS(SUZ12) in such a way that EZH2 wraps around both VEFS and EED and overall holds the entire complex together while connecting the insertion domain of EED to the N-terminal region of VEFS near its SET domain (Jiao and Liu, 2015). Comparison of EZH2 and the minimal PRC2 structures reveal that interaction with EED/SUZ12 rotates the post-SET in such a way that the catalytic channel opens, the cofactor binding site formation is completed, and EZH2 is catalytically competent.

## Reading and Writing H3K27Me3 by EZH2

A structure of the minimal PRC2 complex shows that the complex binds both a stimulating (K27me3) and a [pseudo-]substrate (K27M) H3 peptide simultaneously (Jiao and Liu, 2015). The structure shows that while the substrate H3K27M peptide interacts with the SET domain, the stimulating H3K27me3 peptide binds the β-propeller domain of EED and interacts with the SRM domain of EZH2 (Jiao and Liu, 2015). Structural analysis and enzymatic assays revealed that K27M, observed in glioblastomas, stalls PRC2 activity (Lewis et al., 2013) and spreading of K27 trimethylation due to positioning of arginine 26 in the active site which makes stronger contacts than the wildtype lysine while addition of an R26A mutation to the K27M peptide restores PRC2 MT activity. Interestingly, MT activity using wildtype substrate (H3K27) is increased by over fivefold in the presence of the H3K27me3 peptide which binds EED/SRM(EZH2) (Jani et al., 2019). Accordingly, PRC2 shows lower MT activity on mononucleosome substrates compared to di- or oligo-nucleosome substrates (Yuan et al., 2012). Oligo-nucleosomes reconstituted with short DNA linker (20 vs. 46 and 66 bp) are more robustly methylated by PRC2 indicating that the length of the linker DNA further controls H3K27 methylation. Incubation of PRC2 with an array of peptides collectively spanning H3 1–42 shows enhanced methylation when H3 35–42 peptide was added to the reaction including cases were oligonucleosomes were dispersed (>20 bp linkers). Furthermore, MT assays show that presence of histone H1 positively stimulates PRC2 activity in dinuclesomes (Yuan et al., 2012) suggesting that H1-mediated chromatin compaction stimulates PRC2 activity by providing access to a stimulating H3 from a neighboring nucleosomes.

**FIGURE 3:**
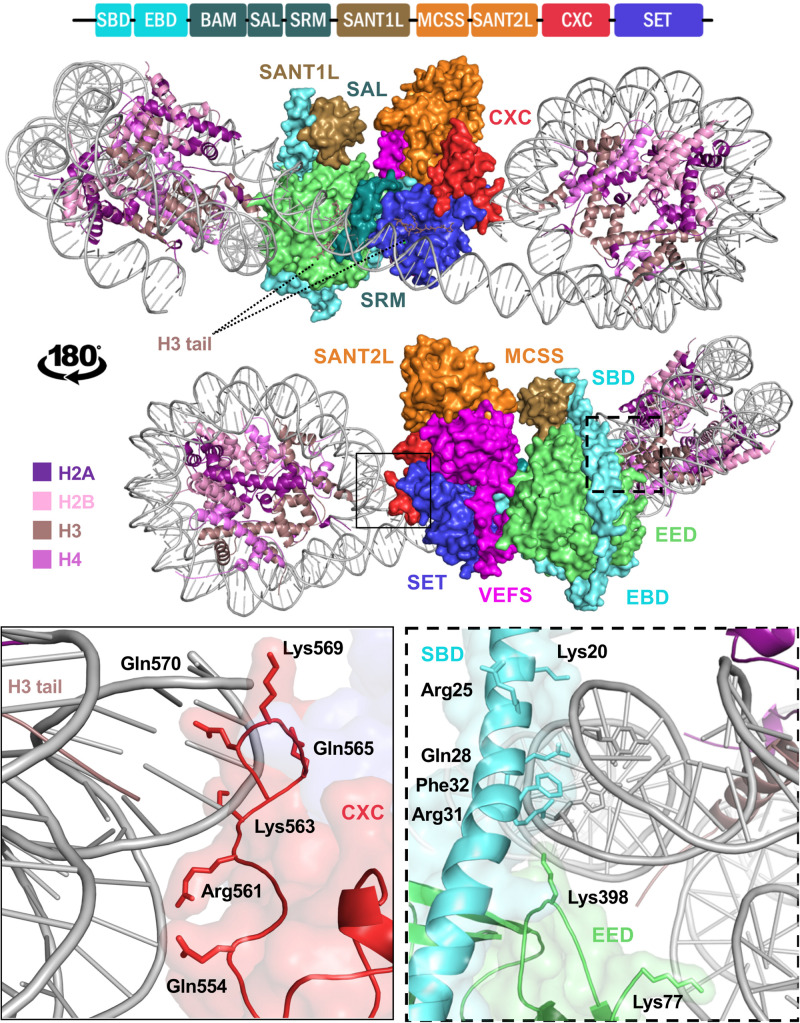
Structure of EZH2 in a minimal PRC2 assembly in complex with an asymmetric di-nucleosome. The schematic at the top represents domain configuration of EZH2. The demonstrations show the same mPRC2:NCP complex from a front and back view. EZH2 domains in the structure are colored according to the linear schematic depiction. The VEFS domain of SUZ12 is colored as magenta and EED is represented as light green. H3 tail with K27M substitution of the substrate nucleosome is shown bound to the substrate groove of EZH2 SET domain (purple blue). The neighboring nucleosome with a modified H3 tail bearing a trimethylated lysine at the position of K27 (K27me3) is shown in a groove between EED and EZH2 SRM domain (teal). Zoomed demonstration of the framed areas on structure is shown at the bottom of the figure. Positively charged and polar residues of EZH2 CXC domain (solid frame) and EZH2 SBD/EED (dashed frame) within proximity to DNA back bone are labeled with their corresponding residue numbers. EZH2 SBD hydrophobic residue is shown in close proximity of DNA.

A cryo-EM structure capturing PRC2 bound to a 35 bp linked dinucleosome provides unique insights into the enzyme complex simultaneously engaging with a pseudo-substrate (K27M) nucleosome and a stimulating (K27me3) neighbor nucleosome (Poepsel et al., 2018). Interestingly, the EZH2 CXC domain makes several contacts with nucleosomal DNA, where the H3 tail extends out of the nucleosome disc (Figure 3). EZH2 SBD also binds DNA at the exit site of the H3 tail but on the neighboring nucleosome (Figure 3). Positively charged and polar residues on the surface of CXC are nearby the DNA backbone. An additional bi-partite sequence, ^491^RKKKRKHR^497^, and ^504^RKIQLKK^510^ in CXC are candidates for DNA interaction; however, these residues could not be modeled in the structure. Similarly, a cluster of polar residues spanning a region of the SBD likely interact with the DNA backbone while an aromatic residue in this region is oriented suitably for intercalating with DNA bases (Figure 3). The area corresponding to EED residues 70 KGKWKSKKCK79 can also potentially bind DNA; however, only residues 77–79 were resolved in the structure of which K79 comes to close contact with DNA backbone. Compared to the crystal structure of PRC2 in the absence of nucleosome, the SET, CXC, and SBD domains are the main components that undergo displacement/tilt after nucleosome binding.

## Discussion

**FIGURE 4:**
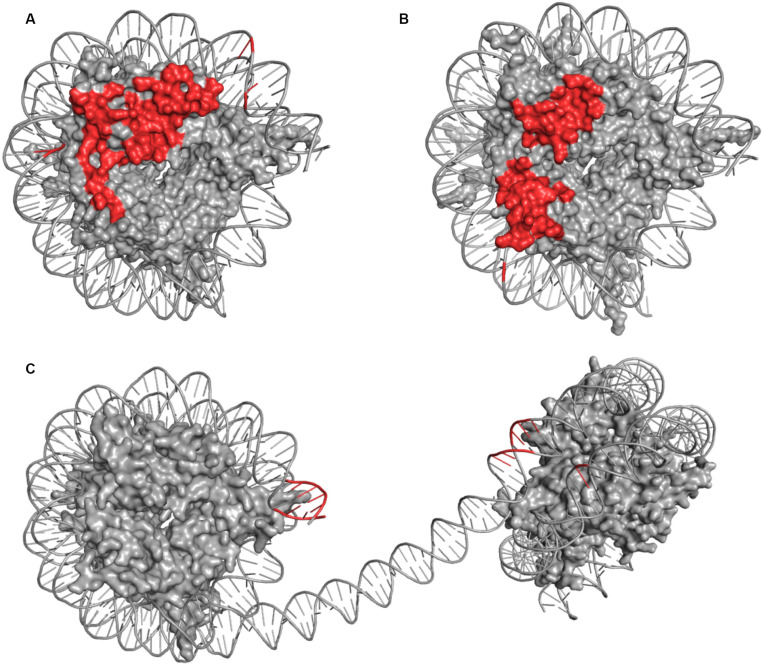
Comparison of DOT1L **(A)**, COMPASS **(B)**, and mPRC2 **(C)** Modes of Engagement with the Nucleosome. The nucleosome discs represent NCP structures captured in the same orientations. The side-by-side comparison shows surface representation of histones inside the nucleosome disc (gray) and cartoons depiction of DNA (gray) highlighting amino acids or DNA bases which come to close contact (≤5 Å) with residues of the associated enzyme or enzyme complex (red). Contact points involving histone tails were omitted due to lack of structural continuity.

Comparative analysis between COMPASS and Dot1L (Figures 4A,B) binding modes of the ubiquitinated nucleosome reveals notable similarities. Both make significant contacts with the surface of the NCP disk and touch each histone protein. Similarly, both make a limited number of contacts with DNA, with, however, differences in the location of these interactions. Dot1L binds DNA adjacent to H3/H2A near histone H3 tail exit site while COMPASS binds DNA near the exit site of histone H4 N-terminus. Also, Dot1L interacts with two distinct regions on the face of the NCP disk, while COMPASS binds a continuous surface. In stark contrast to COMPASS and Dot1L, PRC2 does not contact the surface of the NCP (Figure 4C) but makes several interactions with DNA located at the exit site of the H3 tail on the di-nucleosome.

Together, the cryo-EM structures of Dot1L in complex with the ubiquitinated nucleosome provided pivotal insights into the molecular mechanism underlying Dot1L-mediated methylation H3K79 by histone H2B ubiquitination (Jang et al., 2019; Valencia-Sanchez et al., 2019; Worden et al., 2019; Yao et al., 2019). The cryo-EM structures of COMPASS bound to H2B ubiquitinated NCP uncovered the crucial functions of COMPASS subunits in recognizing different parts of the nucleosome and further the essential functions of SET1 ARM motif in linking H2B ubiquitination and H3K4 methylation. Considering that Dot1L and COMPASS complexes are linked to leukemia, these findings may help in the design of inhibitors that could serve as effective therapeutic agents.

## Author Contributions

All authors equally contributed to the preparation of the manuscript. The sections on Dot1L, COMPASS, and EZH2 were written by MJ, AJ, and HD, respectively. HD also prepared Figure 4.

## Conflict of Interest

The authors declare that the research was conducted in the absence of any commercial or financial relationships that could be construed as a potential conflict of interest.
